# Supplementary material for: H3K4me2 distinguishes a distinct class of enhancers during the maternal-to-zygotic transition
Source: PLoS Biol. 2025 Jul 10;23(7):e3003239. doi: 10.1371/journal.pbio.3003239 (PMC12244696; doi:10.1371/journal.pbio.3003239)
Supplement: S1 File — Uncropped gel images. (PDF) [file pbio.3003239.s014.pdf]

S8B Fig

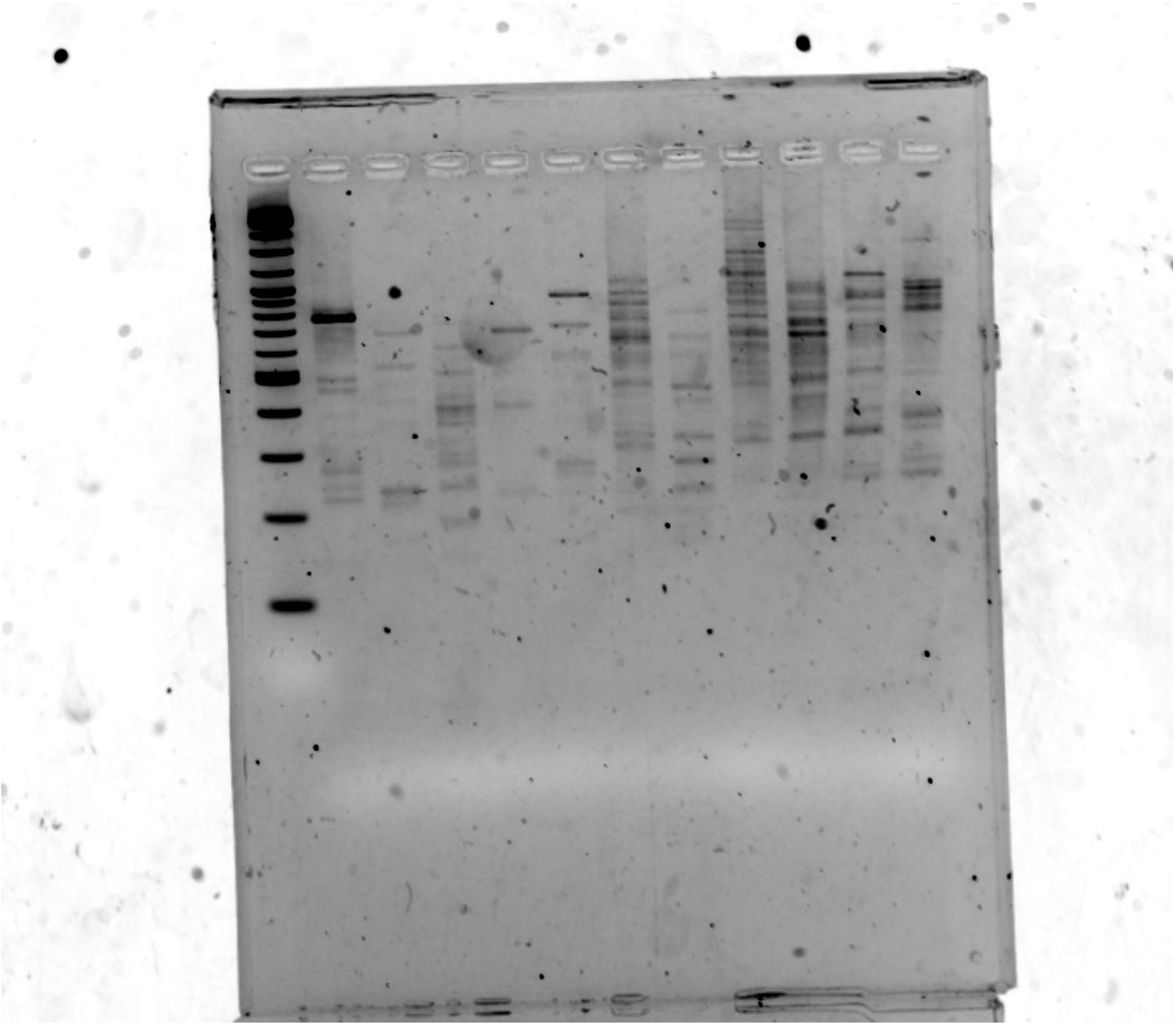

Full gel was used and labeled in the manuscript. Lane 1, 100 bp ladder; Lane 2, WT, remaining lanes = *hapstr1b* enhancer F0 Crispants.

Amersham Imager 600, Blue light, Grayscale capture

S8D Fig (bottom)

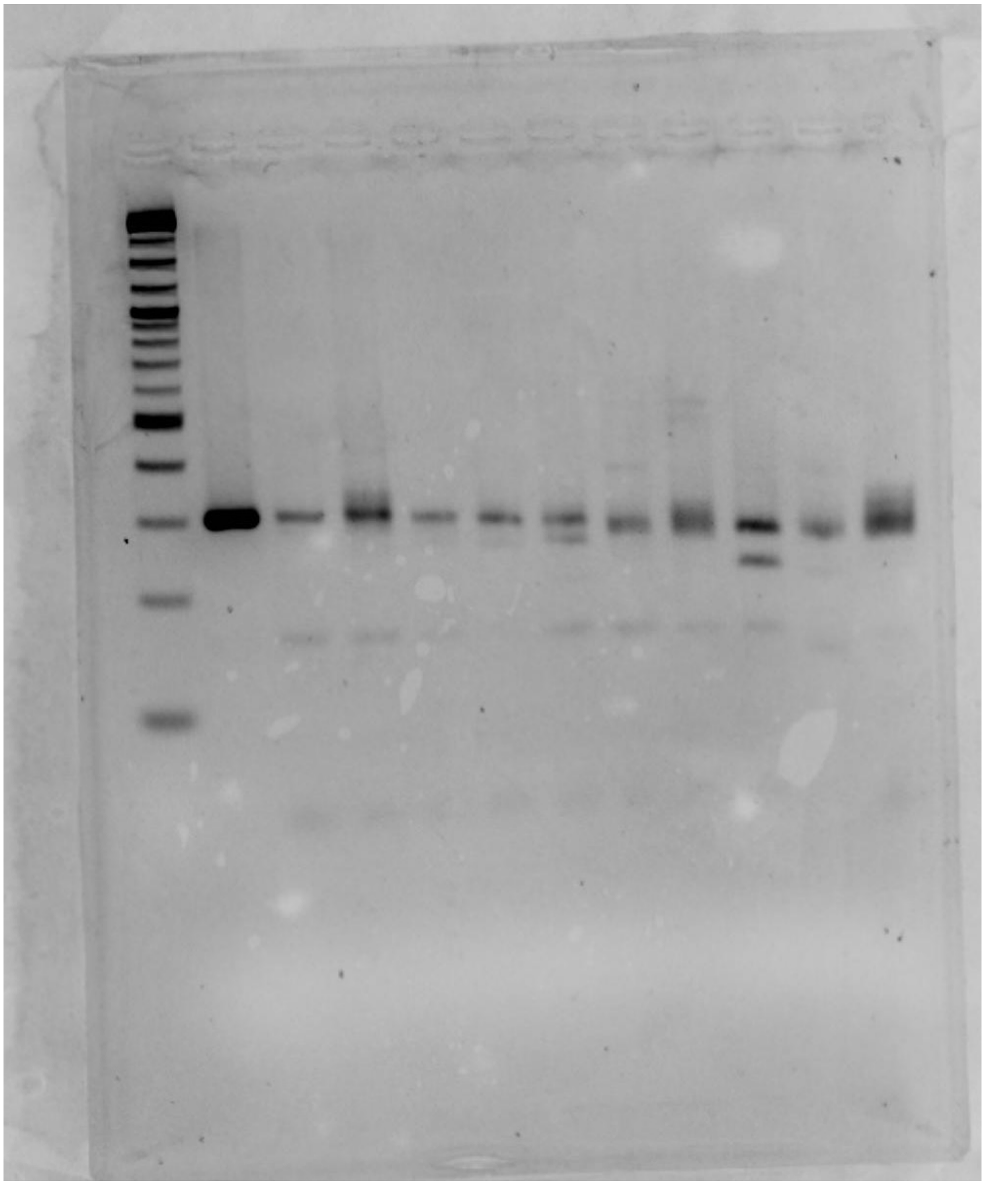

All lanes were used and labeled in the manuscript. Lane 1, 100 bp ladder; Lane 2, WT, remaining lanes = *ier5l* enhancer F0 Crispants.

Amersham Imager 600, Blue light, Grayscale capture

S8D Fig (top)

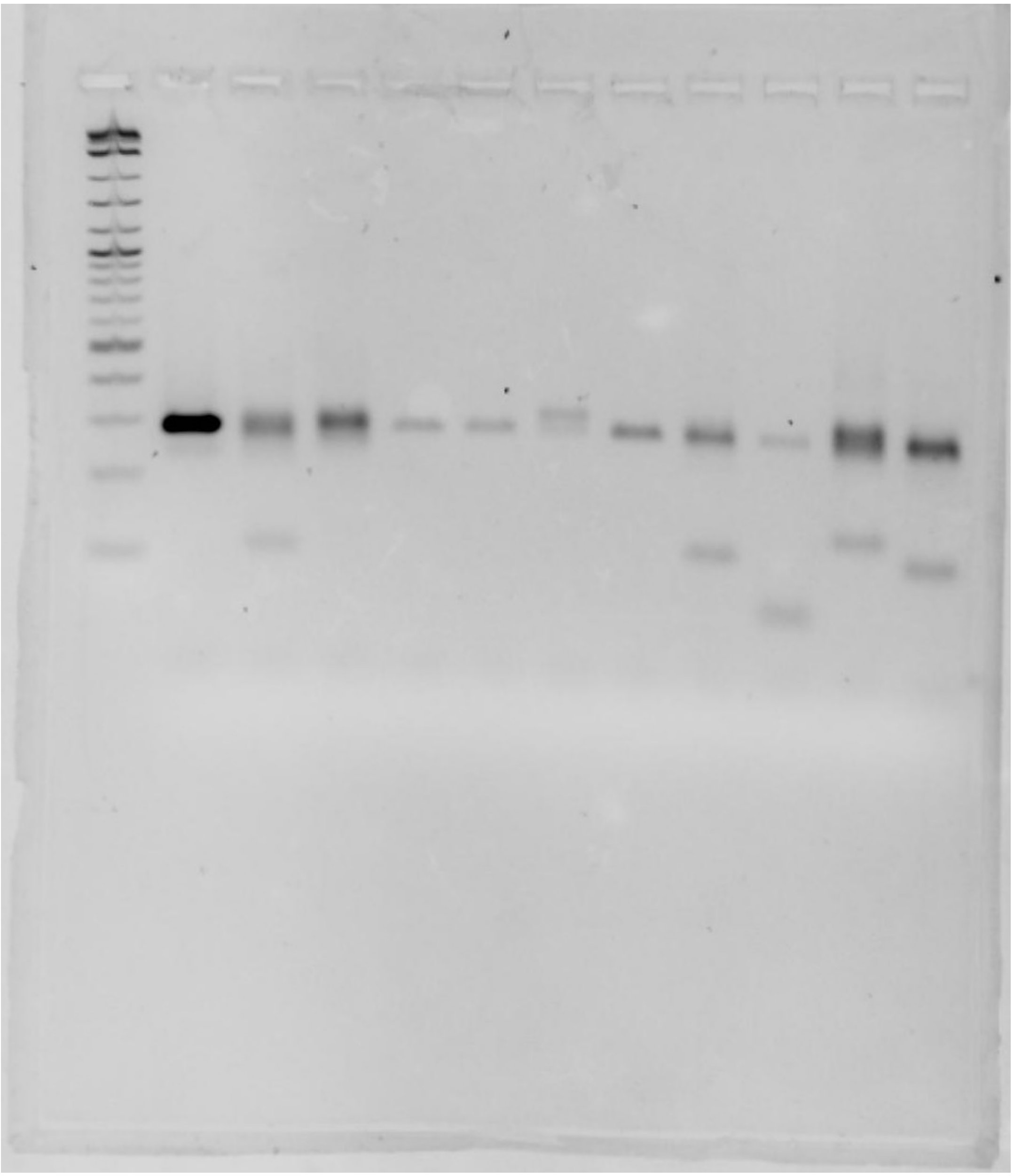

All lanes were used and labeled in the manuscript. Lane 1, 100 bp ladder; Lane 2, WT, remaining lanes = *ier5l* enhancer F0 Crispants.

Amersham Imager 600, Blue light, Grayscale capture

S8E Fig

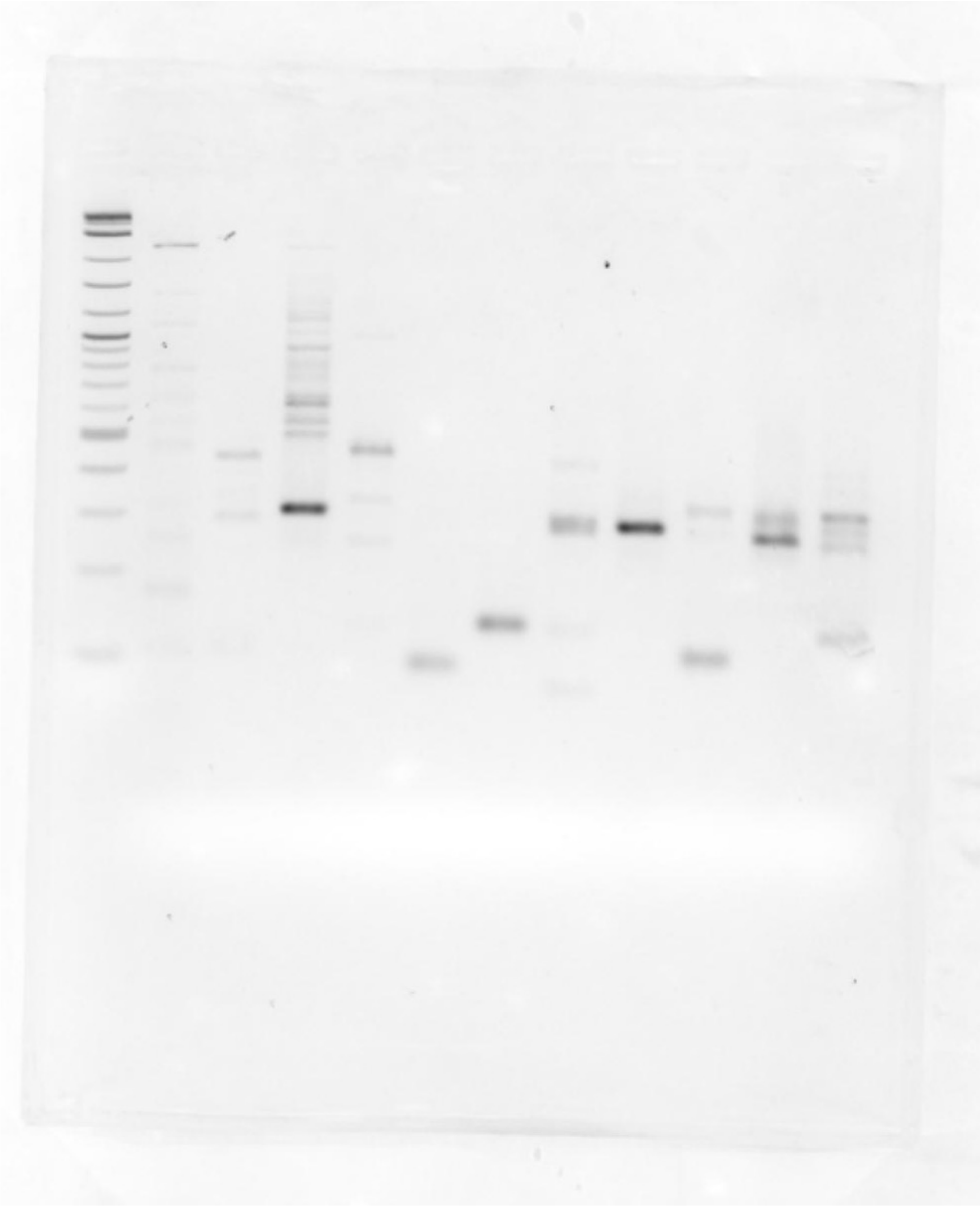

Full gel was used and labeled in the manuscript. Lane 1, 100 bp ladder; Lane 2, WT, remaining lanes = *ier5l* enhancer F0 Crispants.

Amersham Imager 600, Blue light, Grayscale capture
