# Supplementary material for: H3K4me2 distinguishes a distinct class of enhancers during the maternal-to-zygotic transition
Source: PLoS Biol. 2025 Jul 10;23(7):e3003239. doi: 10.1371/journal.pbio.3003239 (PMC12244696; doi:10.1371/journal.pbio.3003239)
Supplement: Attachment — Submitted filename: Hurton24 - Response to reviewers.docx [file pbio.3003239.s017.docx]

Rev. 1:
The manuscript "H3K4me2 distinguishes a distinct class of enhancers during the maternal-to-zygotic transition" by Hurton et al. explores mechanisms for the initial activation of embryonic genes that are not regulated by the canonical trio of zebrafish pluripotency factors. To do this, they employ CUT&RUN to rigorously profile the histone tail landscape of zebrafish chromatin in blastula stage embryos around the time of the onset of the major initial wave of zygotic transcription. Intriguing, they find two subclasses of enhancers that are distinguished by the presence or absence of H3K4me2. Enhancers lacking H3K4me2 are associated with activation by canonical pluripotency factors, while those with H3K4me2 appear to be hypomethylated and to gain accessibility independent of these factors. The manuscript is thorough and the data sets that are generated are likely to provide an important resource for a wide variety of investigators. Arguments for reevaluating H3K4 methylation
are well articulated, and the resulting observation that there are potentially two unique sets of enhancers marked by different H3K4me valencies reveals a new aspect of the mechanisms governing transcriptional activation that will be of interest to researchers working in areas of gene regulation, the maternal-to-zygotic transition, and early cell fate decisions. The fact that accessibility is governed by distinct mechanisms is of interest even though the transcriptional relationships between H3K4me2 enhancers and pluripotency factor independent gene expression were not especially clear cut.


Suggestions for manuscript improvement include:

Line 50. Minor, but I'm not sure the supplied references provide evidence that during the first two hours post-fertilization, chromatin is tightly condensed. Perhaps " has limited accessibility as assessed by ATAC seq" or something of that nature might be more precise?

Indeed! Phrasing has been corrected to: "During the first two hours post fertilization (h.p.f.), genomics assays suggest that embryonic chromatin has limited accessibility and mostly lacks histone modifications..."

Can the authors provide additional information on aligned reads and percent aligned reads in Sup table 1?

We've augmented Supp table 1 to include this information

It would be helpful for the authors to state the number of H3K4me2 marked enhancers, it seems to be a relatively small subset.

The legend of Figure 1 (F) has counts listed; we've also added this line following the RNA-seq filtering (Line XX): "...leaving us with a refined set of 3,268 H3K4me1 enhancers and 681 H3K4me2 enhancers for subsequent analyses"

In figure one and throughout the paper, it could be useful to indicate when data presented are merged data across replicates and the number of replicates.

We've modified the legends of Fig 1, 2, 4, and 5 to indicate that CUT&RUN replicates were pooled and the corresponding Supplementary Figure that shows the individual replicates.

In figure 1. It is not clear to this reviewer what comparisons this statement refers to "H3K4me2 through H2BNTac have significant differences each at P < 1x10-100, and the remaining marks are significant to P < 1x10-30" is there a way to make this clearer?

We've rephrased this: "For each of the remaining marks, significant differences were assessed by individual Kruskal-Wallis tests (H3K4me2, H3K4me3, H3K9ac, H3K27ac, H4K16ac, H2BNTac: P < 1x10^-100^; H3K56ac, H3K26ac, H3K122ac: P < 1x10^-30^)"

In figure 1, it seems enhancers marked by H3K4me2 are also marked by H3K4me1, can the authors be more clear on if they are suggesting these two marks occur together on adjacent nucleosomes? Is it possible embryos are heterogeneous for the two states, with these regions marked by me1 in some cells and me2 in others

Yes, interesting point. We have added a paragraph to the discussion speculating on this. Given that our CUT&RUN is bulk signal over thousands of cells, we think there is likely cell-to-cell locus-to-locus variability in H3K4me1 vs H3K4me2 that arises from enzyme kinetics; though that is certainly not the only possibility.

It could be useful to understand how long these H3K4me2 enhancers persist during development, the title alludes to them in the context of MZT, but might they be more broadly relevant during extended embryogenesis? Their persistence at shield stage seems to raise this possiblity.

Yes, we would speculate that the evolving DNA methylation landscape of the embryo would continue to influence H3K4 methylation degree as development proceeds. We are currently investigating this, though we don't yet have any new insights to report in the current manuscript.

The authors use RNA seq data from white et al to help distinguish promoter and enhancer regions. However, I believe they use a different strain background compared to white et al which could complicate this analysis. Could there be cases where different TSS are used between the two stains in the early embryo? It might be prudent to acknowledge this caviate

We actually performed the RNA-seq analysis on two different datasets (Vejnar et al 2019 and White et al 2017), included only the Vejnar analysis in the original manuscript, but incorrectly cited the White paper. We've now included both (Supp Fig 4B) and mentioned the strains in the legend. The Vejnar dataset uses the same strain as us (TUAB), but the Vejnar and White datasets concur regardless.

Can the authors provide evidence that their triptolide treatment worked?

We added representative photos of leftover embryos comparing triptolide treatment and control to Supp Fig 6B from one of our CUT&RUN experiments. Triptolide-treated embryos failed to initiate epiboly, consistent with inhibited genome activation.

Can the developmental stage for the presented data be included in the legend for experiments in Figure 4?

We've added the stages to the figure.

Can the authors provide more clarity on how methylation proportion was calculated in figure 4? (it could be helpful to break up the data analysis section in the method, it is quite long and dense, making it hard to pull out details, apologies if I missed this) Do K4me2 enhancers show similar CpG density to K4me1 enhancers?

Apologies, we've added subheaders to the Data Analysis section in the Methods. The relevant subsection is "Other chromatin and DNA methylation dataset comparisons," which describes data processing for the DNA methylation using bwa-meth and MethylDackel. We do indeed observe higher CpG density in K4me2 enhancers compared to K4me1 (Supp Fig 6F, G).

Rev. 2:
H3K4me2 distinguishes a distinct class of enhancers during the maternal-to-zygotic transition

General thoughts:
This work explores the mechanisms driving embryonic genome activation in zebrafish during the maternal-to-zygotic transition. While vertebrate pluripotency factors Nanog, Pou5f3, and Sox19b (NPS) are known to pioneer chromatin opening and enable gene activation, some genes are transcribed without these factors, indicating the presence of alternative regulatory mechanisms. Using CUT&RUN to profile histone modifications, the authors identify two types of enhancers: those marked by H3K4me2, which are epigenetically bookmarked and function independently of NPS, and those lacking H3K4me2, which depend on NPS for activation. These findings reveal dual pathways for enhancer activation in early embryonic development.

Overall, the paper presents an interesting model distinguishing two classes of enhancers and leverages multiple experimental modalities to explore this concept. At certain points it does lack an overall view of the data created for the analysis. It can also benefit from some more in depth analysis, like using the staged ATAC-seq to explore these regions and potentially GO-enrichment analysis on the genes near the two different classes to look at if there is any biological trend to NPS-independent regulated genes. The enhancer reporter assay could also be expanded, with examples of promoter regions from the two sub-populations.

We have added analyses as suggested by the reviewer below. Additionally, we have added a GO enrichment analysis (Supp Fig 7E) -- no terms are significant after multiple test correction that distinguish H3K4me1 enhancer - associated genes vs H3K4me2 enhancer - associated genes, though there is a trend toward embryo-specific functions among the H3K4me1 enhancer-associated genes and chromatin binding for H3K4me2.

Major comments:

Figure 1 & Supplementary Figure 1:
* It would be beneficial to include a more comprehensive PCA analysis genome-wide across different replicates and histone modifications. This would provide a clearer picture of how these factors influence the data and allow for a more robust interpretation of the results.

We have now included a PCA across individual replicates of all the histone modifications at dome stage (Supp Fig 1A-C) (and across time, see below), yielding similar trends to the pooled PCA. Overall signal across all marks still defined PC1, and H3K4 methylation differences still drive most of the remaining variation.

* The classification of certain promoters as "enhancer-like" is not entirely convincing. It is unclear if these are genuinely enhancer-like or simply inactive promoters. A more thorough analysis, perhaps utilizing ATAC-seq data across developmental stages (as shown in Liu et al., 2018), could clarify whether these promoters are dynamically regulated.

Apologies, it was not our intent to imply any actually enhancer-like function for the promoters that computationally clustered with the majority of bona fide enhancers. In the text, we do suggest that these are indeed likely inactive promoters. However, to avoid confusion, we have now renamed the promoters "High H3K4me2/3 promoters" and "Low H3K4me2/3 promoters" after their defining characteristic.

* Figure 1SE is currently not well explained in the results section and appears somewhat disconnected from the overall message. Providing more context and a clearer rationale in both the figure legend and the results would make it easier for readers to grasp its relevance.

We've rewritten the results to better contextualize Supp Fig 1E-F (now Supp Fig S2 C-D) and augmented the legend.

Results section: "Finally, we found that the core globular acetylation marks H3K56ac, H3K64ac, and H3K122ac, which have been linked to unique enhancer subtypes in other systems (Pradeepa et al., 2016; Tan et al., 2013), do not seem to differentiate our two enhancer classes (Fig 1F, G, S2A Fig). H3K56ac does contribute to the fourth principal component, but we did not discover any new enhancer subclasses in zebrafish induced by this or the other core acetylation marks (S2C, D Fig)."

S2 Fig legend: "(C) Heatmaps of regions stratified by the fourth principal component (PC4) of the pooled PCA, which H3K56ac strongly contributes to. H3K56ac has been proposed to be an Oct4-associated enhancer mark in mouse ES cells (Tan et al, 2013). PC4 high = ATAC-seq regions with PC4 values greater than the 1 standard deviation from 0, PC4 low = less than -1 * standard deviation. Enhancers stratified by PC4 have differential H3K56ac, but do not differ in other enhancer-associated marks. Parallel heatmaps of Nanog, Pou5f3 (Oct4 homolog), and Sox19b ChIP-seq coverage (data from Miao et al, 2022) demonstrate no correlation with PC4 / H3K56ac. No diferences are seen among promoters either. Thus, H3K56ac likely does not define a distinct regulatory element class in zebrafish embryos. (D) Heatmaps of regions enriched for H3K122ac (>2-fold over IgG), stratified by H3K27ac co-enrichment (<1.25-fold or >1.5-fold), showing very few regions marked by H3K122ac without H3K27ac. Pradeepa et al 2016 identified a subclass of H3K122ac+/H3K64ac+/H3K27ac- enhancers in mouse, but enhancers with that pattern do not seem to be prevalent in zebrafish embryos."

Figure 2 & Supplementary Figure 2:
* Like the suggestion for Figure 1, a PCA analysis for these histone marks could provide additional insights, particularly regarding whether developmental progression is captured.

We've included all-stage PCAs in Supp Fig S3C-E, including one that maps the trajectory of H3K4 methylation over the three stages and shows distinct activation paths for H3K4me1 enhancers versus H3K4me2 enhancers (Supp Fig S3D-E)

* There seems to be a lack of signal in the data for the Invitrogen antibody used in H3K4me1, which raises the question of whether Invitrogen data was used for analysis.

We have better clarified the use of the two different H3K4me1 antibodies at 1K-cell stage in the relevant figure panel legends and in the text. As we noted, only the Active Motif antibody yields any elevated signal at 1K-cell stage, suggesting that overall H3K4 methylation levels are low. We might speculate that since the Invitrogen antibodies are more stringent, we could be measuring some degree of pan H3K4 methylation with the Active Motif antibody. This does not affect our interpretation for the enhancers, since only the dome-stage H3K4me2 enhancers have Active Motif H3K4me1 signal anyway.

Figure 3 & Supplementary Figure 3:
* I have a general concern regarding the classification of enhancers as "promoter-like" in Figure 1 based on PCA data. From the limited sampling, there does not appear to be a strong indication that "promoter-like" enhancers have a higher potential to act as promoters than typical enhancers. A more detailed justification or additional data would be helpful.

Again, we had no intent to imply promoter-like functionality for the enhancers, and have changed the nomenclature to avoid this interpretation.

* Including examples of typical promoters and "enhancer-like" promoters would be very informative. Are these promoters inactive in general, or do they exhibit enhancer-like activity? This comparison could help clarify the functional differences between these two categories.

* Figure S3D is quite confusing and is not adequately discussed in the results section. Additionally, it seems to imply that promoters would produce similar signals as enhancers, raising questions about whether this assay is suitable for distinguishing enhancer activity from promoter activity.

We have now included some additional promoter reporters from each of the two promoter categories (Supp Fig 5E-F). The zebrafish literature suggests that proximal promoter sequence is generally insufficient to drive reporter gene expression, and indeed we only observe very faint (below the threshold for our original enhancer assays) fluorophore signal for promoter reporters...with the exception of arhgap18, the reporter in question from original Supp Fig 3D.

The history of the arhgap18 element was that we originally thought it was an intergenic element based on Ensembl gene annotations, but later found that it overlapped a predicted RefSeq gene TSS, and thus included it as a promoter element in the original manuscript. In retrospect, indeed it is odd that it shows such strong enhancer activity -- but, this is not a general trend, given the weak enhancing potential for the new promoter reporters. We now speculate in the results section that the arhgap18 element could be dual functioning. The gene itself is not expressed until much later in development (Supp Fig 5G), so it's possible that the element could instead have enhancer capacity in early development.

* It would also be helpful to display the signals for all colors in the panels.

We have redesigned Supp Fig 5 to include all 3 channels in black and white (some reporters were reimaged to now have images for all 3 channels).

Figure 4 & Supplementary Figure 4:
* To further differentiate between these two classes of enhancers, performing a footprinting analysis using TOBIAS would be a valuable addition. Since ATAC-seq data is already available at the relevant developmental stages, this analysis could provide additional insights into differential potential transcription factor binding events and enhancer function across the two classes of enhancers.

We have performed TOBIAS on the enhancer classes (Supp Fig S6D). The analysis shows enrichment of some additional factors not among the top Homer hits.

Minor comments:

Figure 1 & Supplementary Figure 1:
o The data presented in panel A could more clearly indicate that it is from the dome stage to clarify the specific developmental stage being analyzed.

We've reiterated the stage in the legend and added "Dome stage" to Fig 1D, F and Supp Fig 1, 2

o The introduction of terms such as "promoter-like enhancers," "typical promoters/enhancers," and "enhancer-like promoters" can be confusing. It would be easier on the reader to use a consistent terminology throughout the paper. For instance, the histone modification-based naming convention (e.g., "H3K4me1 enhancers" and "H3K4me2 enhancers") could be introduced early on and used consistently.

We've standardized the nomenclature to only use "H3K4me1 enhancers" and "H3K4me2 enhancers"; and "High H3K4me2/3 promoters" and "Low H3K4me2/3 promoters"

Figure 3 & Supplementary Figure 3:
o The legend for Fig. 3S (panel A) is missing a reference to the relevant Supplementary Table number.

Oops, we've corrected the table call

o The BFP signal is hard to see. Using a more distinct color overlay (e.g., yellow) would enhance visibility and make the data more interpretable.

We false colored the BFP to cyan and included grayscale versions of all three channels.

Figure 4 & Supplementary Figure 4:
o The discussion in lines 219-220, stating that no motifs unify H3K4me2, is somewhat vague. Comparing panel SD to panel SB, it doesn't appear that motifs like Nanog, Pou5f3, or Sox19b unify H3K4me1 more strongly than H3K4me2..

This is a fair point -- we tend to treat Nanog, Pou5f3, and Sox19b binding as a singular phenomenon (and this relates to their capacity to complex), but indeed their individual contributions may only apply to a subset of enhancers. We've moderated the phrasing, and in the Discussion clarified: "Although H3K4me1 enhancers seem to be unified by their need for NPS pioneer factor binding, we do not yet know what, if any, transcription factor regulatory logic is shared by H3K4me2 enhancers. Several enriched transcription factor binding motifs are found in various subsets of H3K4me2 enhancer regions (Supp. Fig 6D, E), so it could be that a diverse collection of factors each bind a subset of different H3K4me2 enhancers."

o In panel F, some of the text is cut off.

We've verified that Fig 4F and Supp Fig S6H (formerly Supp Fig 4F) are complete

Figure 5 & Supplementary Figure 5:
o It might be better to call open regions using macs3 to make use of hmmratac for potentially more robust analysis.

Yes, good suggestion for the future. Comparing the hmmratac algorithm to macs2, it seems that macs2 false positives tend to lack histone modifications (so, they would not enter into our analysis, which centers on histone modifications). So the net effect on the current analysis using macs2 would likely be under-calling open regions and thus underestimating the number of putative enhancers.
